# Active Plasmonics with Responsive, Binary Assemblies of Gold Nanorods and Nanospheres

**DOI:** 10.3390/nano11092296

**Published:** 2021-09-03

**Authors:** Piotr Szustakiewicz, Natalia Kowalska, Maciej Bagiński, Wiktor Lewandowski

**Affiliations:** Faculty of Chemistry, University of Warsaw, 1 Pasteura St., 02-093 Warsaw, Poland; p.szustakiewicz@chem.uw.edu.pl (P.S.); n.kolsut@student.uw.edu.pl (N.K.); mbaginski@chem.uw.edu.pl (M.B.)

**Keywords:** gold nanoparticles, self-assembly, plasmonics, plasmon coupling, nanorods, reconfigurable, binary, thin films

## Abstract

Self-assembly of metal nanoparticles has applications in the fabrication of optically active materials. Here, we introduce a facile strategy for the fabrication of films of binary nanoparticle assemblies. Dynamic control over the configuration of gold nanorods and nanospheres is achieved via the melting of bound and unbound fractions of liquid-crystal-like nanoparticle ligands. This approach provides a route for the preparation of hierarchical nanoparticle superstructures with applications in reversibly switchable, visible-range plasmonic technologies.

## 1. Introduction

Strong and controllable light–matter interactions of metallic nanoparticles (NPs), enabled by localized surface plasmon resonances, make them ideal candidates for the construction of compact, optically active elements in future generations of optoelectronic devices [1,2,3,4,5]. Such materials offer a good degree of optical property tunability by controlling the composition, size, shape and packing of NPs [6]. However, further broadening the range of applications requires research on materials exhibiting complex plasmon coupling. One way is obtaining unconventional phases [7] of NPs in binary and ordered systems. In this way, exploration of shape and ligand complementarity of NPs, inspired by the periodic nature of crystal lattices of common materials [8], can e.g., result in strikingly different optical properties in layered, binary assemblies, depending on the arrangement of layers [9]. Another approach to advance coupled plasmonic materials relies on the fabrication of active plasmonic NP systems that can be tuned or reversibly switched by an external stimulus [10]. Structural reconfiguration in assembled, active materials translates to controllable optical properties required e.g., for switchable metamaterials [11]. Combining the two strategies, i.e., achieving binary and active plasmonic systems within a single composite, is an emerging trend allowing us to benefit from the unique characteristics of binary and active NP assemblies. However, the fabrication of binary active materials is extremely challenging.

Until now, the successful realization of reversible switching of structural and optical properties in binary assemblies has been achieved only in aqueous dispersions of DNA-coated NPs [12]. For example, it has been shown that in assemblies of 11 nm diameter and sub 1 nm diameter, DNA-coated Au NPs, smaller spheres were able to move within the crystal lattice formed by larger NPs, indicating they adopt a dynamic, electron-like behavior [13]. However, translating this progress into the development of devices requires the development of cheap and scalable fabrication methods of active and binary NP systems working in thin-film configuration (without solvent). Achieving this goal is challenging from a material point of view. A recently developed approach, enabling the efficient formation of reconfigurable plasmonic films, relies on covering NPs with liquid-crystal-like (LC-like) ligands. Thermo- and optoresponsive characteristics of the ligands enable the reconfiguration of NP assemblies, allowing for dynamic control over, e.g., epsilon-near-zero [14] and chiral [15] plasmonic properties, although this approach [16] has been applied exclusively in unary assemblies of NPs.

The goal of this work is to fabricate periodic binary assemblies of NPs with reversibly switchable symmetry in the thin-film state, leading to controllable plasmonic coupling in the visible range. To achieve this goal, we explored the LC-based strategy of self-assembly. An LC-like ligand was introduced to the surface of nanospheres (NSs) and nanorods (NRs) and added as an unbound fraction to thin-film assemblies to increase the fluidity of the NRs and enable reversible reconfigurability. Temperature-controlled self-assembly of these components resulted in the thin films exhibiting reversibly switchable, thermally responsive symmetry. By varying the content of NSs in binary assemblies, we were able to tune the optical properties of the films within the visible range. We combined structural and optical data to fully explain the observed phenomena.

## 2. Materials and Methods

### 2.1. Materials for NP Synthesis

Tetrachloroauric acid (HAuCl_4_, ≥99%), hexadecyltrimethylammonium bromide (CTAB for molecular biology ≥99%), 1-decanol (*n*-decanol, 98%), silver nitrate (AgNO_3_, ≥99.0%), hydrochloric acid (HCl, 37%), sodium borohydride (NaBH_4_), L-ascorbic acid (AA, ≥99%), tetrahydrofuran (THF, anhydrous, ≥99.9%), dodecanethiol (DDT, ≥98%), toluene (anhydrous, ≥99.8%), dodecylamine (≥99%), cyclohexane (99.5%), formaldehyde (37 wt% in H_2_O, 10–15% methanol as stabilizer) and ethanol (95%) were purchased from Merck (Darmstadt, Germany). All chemicals were used without further purification. Milli-Q water was used in all experiments.

### 2.2. Materials for Organic Synthesis

All reagents and solvents were obtained from Sigma-Aldrich (St. Louis, MO, USA). Before use, solvents were dried over activated molecular sieves for 48 h. Substrates were used without purification. All reactions were carried out under nitrogen conditions in dried glassware, ensuring efficient magnetic stirring. Purification of reaction products was carried out by column chromatography using RushanTaiyang silica gel 60 (230–400 mesh) at atmospheric pressure or by crystallization. Analytical thin-layer chromatography (TLC) was performed using a Silica Gel 60 ÅF254 (Merck, Darmstadt, Germany) precoated glass platter (0.25 mm thickness) and visualized using iodine vapor and/or UV lamp (254 nm). Yields refer to chromatographically and spectroscopically (^1^H NMR) pure materials. The ^1^H NMR and ^13^C NMR spectra were recorded using 500 MHz NMR Varian Unity Plus. Proton chemical shifts are reported in ppm (δ) relative to the internal standard—tetramethylsilane (TMSδ = 0.00 ppm). Carbon chemical shifts are reported in ppm (δ) relative to the residual solvent signal (CDCl3, δ = 77.0 ppm).

### 2.3. Gold Nanosphere Synthesis (NS)

The synthesis of gold nanoparticles was carried out according to the method reported by Chen and Wang [17]. A 0.75 g amount of dodecylamine was dispersed in 25 mL of cyclohexane, and 6 mL of 37% formaldehyde solution was added. The mixture was stirred for 20 min at 25 °C. Afterward, the mixture was centrifuged (5000 rpm over 5 min) in order to separate the organic phase from the aqueous phase, which was collected and washed twice with water. Then, 10 mL of an aqueous solution of HAuCl_4_ (1 g of HAuCl_4_ in 250 mL H_2_O) was added under vigorous stirring and left for 40 min. Afterward, the organic phase containing nanoparticles was separated through centrifugation (5000 rpm over 5 min), and 0.5 mL of the dodecanethiol was added. The reaction was stirred overnight. Then, nanoparticles were precipitated by the addition of ethanol, centrifuged (5000 rpm over 5 min), collected, redispersed in a small amount of cyclohexane, and the procedure was repeated. Finally, purified NSs were dissolved in cyclohexane.

### 2.4. Gold Nanorod Synthesis (NR)

Synthesis of gold nanorods was carried out according to the seed-mediated growth method reported by González-Rubio et al. [18]. For the preparation of the seeds, 200 μL of 50 mM HAuCl_4_ and 100 μL of 100 mM ascorbic acid were added to 20 mL of 50 mM CTAB and 13.5 mM decanol solution. Afterward, 800 μL of freshly prepared 20 mM NaBH_4_ was injected under vigorous stirring. The seeds solution was left for 60 min to remove the excess borohydride. For NR synthesis, 18 mL of seeds solution was added under vigorous stirring to the solution containing 300 mL of 50 mM CTAB and 13.5 mM decanol solution, 2.4 mL of 10 mM AgNO_3_, 3 mL of 50 mM HAuCl_4_, 21 mL of 1 M HCl, and 3.9 mL of 100 mM ascorbic acid. The mixture was left undisturbed for at least 4 h. Before further modification, NRs were centrifuged two times (14,000 rpm over 45 min) to remove the excess reactants and redispersed in 10 mM CTAB solution.

### 2.5. NS Functionalization

A 5 mg amount of the obtained NSs were precipitated from the cyclohexane solution through the ethanol addition. The precipitate was centrifuged (5000 rpm over 5 min) and redispersed in 500 μL of a toluene solution containing 5 mg of LC ligand in an ultrasonic bath. Afterward, the mixture was left under mild stirring overnight. Then the excess ligand was removed through centrifugation (5000 rpm over 5 min) and modified NSs were redispersed in toluene to a concentration of 3 mg/mL.

### 2.6. NR Functionalization

NRs were functionalized analogously to our previous work [19]. Briefly, the obtained NRs were centrifuged (14,000 rpm over 45 min) and redispersed in LC—DDT solution in THF in an ultrasonic bath. In a typical procedure, the molar ratio of Au (0) to DDT to L was 1:1:1. The mixture was left under mild stirring overnight. Afterward, the excess ligand was removed through centrifugation and modified NR were redispersed in toluene to a concentration of 3 mg/mL.

### 2.7. Binary Systems Preparation

For NR-L preparation, 30 μL of L-toluene solution (1 mg/mL) was added to 100 μL of NR toluene solution (3 mg/mL). The mass ratio of L to NR was 1:10. A binary system comprising NR, NS and unbound L was prepared in two NR to NS particle ratios—1:5 (NR-NS-L1) and 1:20 (NR-NS-L2). For NR-NS-L2 preparation, 29.4 μL of L-toluene solution (1 mg/mL) was added to 98 μL of NR toluene solution (3 mg/mL) and 60 μL of NS toluene solution (3 mg/mL). For NR-NS-L1 mixture preparation, 29.4 μL of L-toluene solution (1 mg/mL) was added to 98 μL NR toluene solution (3 mg/mL) and 15 μL of NS toluene solution (3 mg/mL). The mass ratio L to NRs in each sample was 1:10. To perform TEM measurements, binary mixtures were drop casted on a TEM grid (∼3 μL). Subsequently, the samples were heated to 120 °C and cooled to 30 °C with a precisely controlled rate of 5 °C/min. For SAXRD measurements, samples were prepared as thin films on Kapton tape (∼3 μL). Measurements were carried out in the range of temperatures from 30 °C to 120 °C every 10 °C. For UV–vis measurements, binary mixtures were drop casted on microscope slides (∼5 μL). Measurements were carried out at 120 °C and 30 °C in a wavelength range from 400 nm to 1000 nm.

### 2.8. Other Methods

The cooling/heating process of the samples was controlled using the FTIR600 SP Linkam stage with T96 LinkPad system controller, available at the University of Warsaw. For structural analysis of the obtained materials, transmission electron microscopy was used: TEM model JEM-1011 (JEOL, Tokyo, Japan) equipped with a model EDS INCA analyzer (Oxford, UK), in the Electron Microscopy Platform, Mossakowski Medical Research Centre, Polish Academy of Science, Warsaw. SAXRD measurements were performed with a Bruker Nanostar system (Cu Kα radiation, parallel beam formed by cross-coupled Goebel mirrors and a 3-pinhole collimation system, VANTEC 2000 area detector, Bruker, Billerica, MA, USA). Spectroscopy measurements in the UV–vis range were performed using GENESYS 50 UV-Vis spectrophotometer (Thermo Fisher Scientific, Waltham, MA, USA), available at the University of Warsaw.

## 3. Results and Discussion

**Figure 1:**
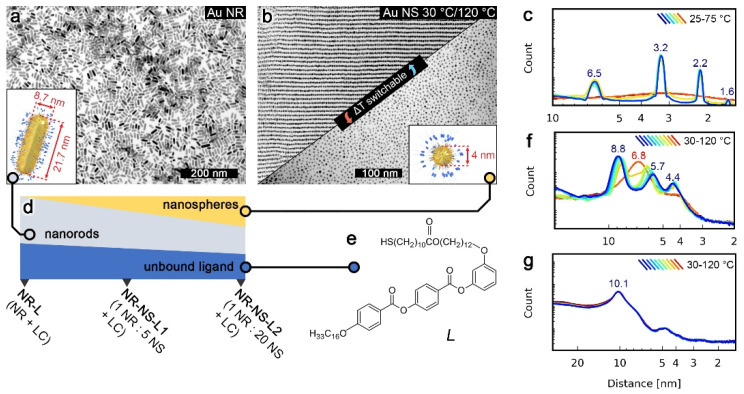
Components and design of the tested NP systems. (**a**) TEM image and a model (inset) of Au nanorods (NRs). (**b**) TEM micrographs and a model (inset) of Au nanospheres (NSs); the upper and lower TEM images were recorded for samples quenched at 30 and 120 °C, respectively, evidencing a thermally dependent structure of NSs in the neat state, in contrast to a static NRs assembly. (**c**) 1D small-angle XRD patterns of the L ligand at different temperatures. (**d**) Graphical scheme indicating composition (by mass-%, unbound ligand bar is magnified 10× times) of assemblies studied. (**e**) L ligand structure. (**f**) 1D small-angle XRD patterns of Au NSs at different temperatures. (**g**) 1D small-angle XRD patterns of Au NRs at different temperatures; note that the pattern does not change with the temperature.

The LC-like ligand used in this study (L, Figure 1e) was designed to have a rigid, aromatic core and two aliphatic chains, a common architecture for thermoresponsive LC ligands. The synthetic path leading to L is shown in Figure S1. At low temperature, L forms a layered crystal and melts to the isotropic phase at ~60 °C (Figure 1c). NPs covered with a mixed monolayer of L and dodecanethiol (DDT) were prepared—Au NRs (8.7 × 21.7 nm, Figure 1a) and Au NSs (∅ = 4 nm, Figure 1b).

### 3.1. Structural Properties of NSs and NRs

**Figure 2:**
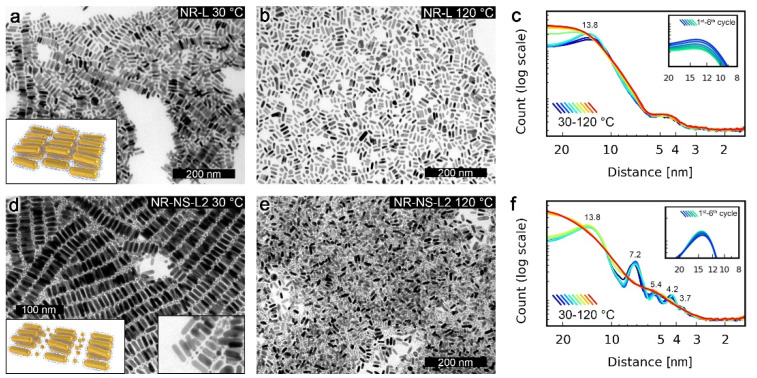
Measurements of NP assemblies with unbound L. (**a**) TEM image of NR-L heated to 120 °C and slowly cooled down to 30 °C, revealing the tendency of NRs to form side-to-side assemblies. (**b**) TEM image of NR-L quenched at 120 °C, revealing a lack of long-range ordered structure. (**c**) Temperature evolution of SAXRD diffractograms of NR-L. The inset presents a selected XRD peak observed at 30 °C after consecutive heating/cooling cycles, confirming the reversibility of the thermally induced reconfiguration of the assemblies. (**d**) NR-NS-L2 heated to 120 °C and slowly cooled down to 30 °C, revealing the tendency of NRs to form side-to-side assemblies. (**e**) TEM image of NR-NS-L2 quenched at 120 °C. Note the intercalation of NR layers by NS. (**f**) Temperature evolution of SAXRD diffractograms of NR-NS-L2 systems. The inset presents a selected XRD peak observed at 30 °C after consecutive heating/cooling cycles, confirming the reversibility of the thermally induced reconfiguration of the assemblies. Note the more precise overlap of diffractograms in consecutive switching cycles, suggesting higher stability of binary vs. unary (shown in (**c**)) assemblies of NRs.

The structural reconfigurability of NSs and NRs was first assessed in their unary films, based on diffraction patterns collected using temperature-dependent small-angle X-ray diffraction (SAXRD), as well as transmission electron microscopy (TEM) images of samples quenched at different temperatures. These analyses confirmed that NSs exhibit a phase transition between long- and short-range ordered phases at ~100 °C (Figure 1b,f). Additional TEM micrographs of high- and low-temperature NS assemblies are shown in Figure S3. In the case of NRs, XRD data revealed no phase transition (Figure 1g). A detailed structural description of NSs and NRs systems is given in Supplementary Note 1. The contrast between the static nature of NRs vs. the switchable nature of NSs seemed to originate from the higher metal to organic volume ratio of NRs [11,20] than NSs. Thus, motivated by a recent report on the role of unbound ligands, [21] we tested if the addition of a small portion (~10% by mass) of L to NRs (i.e., preparation of sample named NR-L) could lead to achieving reconfigurability. Below 90 °C, SAXRD of NR-L revealed a single, broad peak corresponding to ~13 nm periodicity (Figure 2c). Above 90 °C, the peak broadened, suggesting a shortening of the correlation length. TEM images of NR-L further confirmed the switchable nature of the material. A sample heated to 120 °C and cooled down slowly to 30 °C revealed layers of side-to-side (S-S) arranged NRs, as well as stacking of layers (Figure 2a), while sample quenched at 120 °C showed loosely packed side-by-side assemblies of NRs (Figure 2b).The switchability of NR-L, as well as an increased S-S NR gap (NR-L vs. NR, Figure S4), can be attributed to unbound L molecules intercalating the NRs. This fact highlights the chemical compatibility between L-coated NPs and L and the crucial role played by unbound ligands.

### 3.2. Structural Properties of NR-NS Binary Systems

Motivated by the switchability of NR-L, we prepared binary samples of NRs and NSs doped with (10% by mass) unbound L, named NR-NS-L1 and NR-NS-L2 (Figure 1d), with 1:5 and 1:20 NRs/NSs particle ratios, respectively. SAXRD measurements of NR-NS-L2 revealed that this binary composite formed long-range ordered structures at low temperatures and melted to an isotropic phase at 100 °C (Figure 2f). More specifically, diffractograms collected at 30 °C revealed signals characteristic to S-S-arranged NRs (~14 nm periodicity) and partially isotropic NS assemblies. XRD measurements of NR-NS-L1 revealed similar signals (Figure S2). TEM images collected for the NR-NS-L2 sample cooled down slowly from 120 °C (Figure 2d) revealed binary structures comprising alternating layers of hexagonally arranged NRs and non-long-range ordered NSs, in agreement with SAXRD measurements. In contrast, TEM images collected for samples quenched at 120 °C did not reveal long-range ordered structures, corresponding to SAXRD data collected at 120 °C. Additional TEM micrographs are shown in Figures S5 and S6. A similar superstructure was previously observed by Murray et al. for nonplasmonic NPs [22]. It is worth noting that our materials exhibited reversible reconfigurability, as evidenced by SAXRD diffractograms collected in six consecutive heating/cooling cycles (Figure 2f, inset). It is also worth highlighting the role of NSs at elevated temperatures; above their melting point, NSs seem to behave as a solvent for NRs, as indicated by the TEM images of thin parts of the sample quenched at 120 °C (Figure 2e).

### 3.3. Switchable Plasmonic Properties

**Figure 3:**
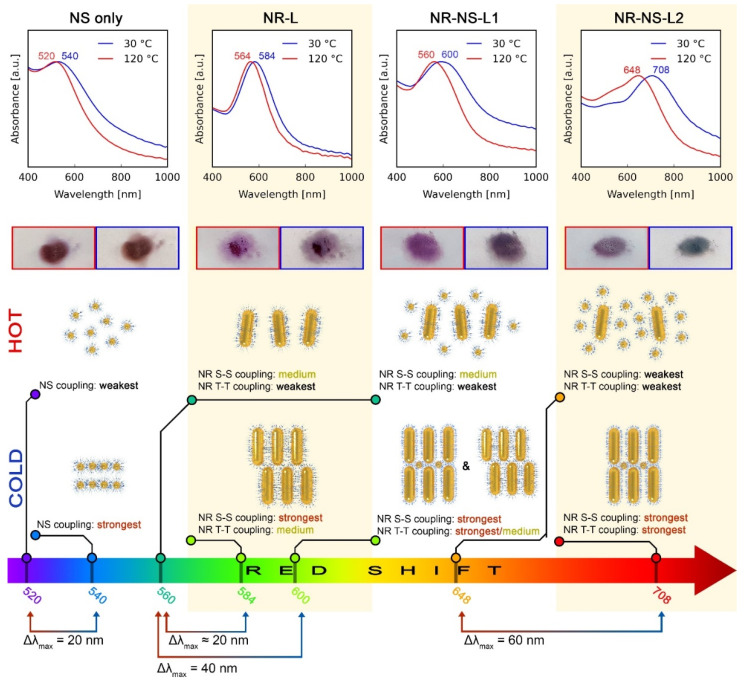
Far-field optical properties of NS, NR-L, NR-NS-L1 and NR-NS-L2 films (consecutive columns). Consecutive rows represent: (1st row) UV–vis spectra of a given material at 30 and 120 °C (blue and red curves, respectively). (2nd row) Optical images of the films on a glass substrate at 30 and 120 °C (the longer side of the rectangle represents 10 mm). (3rd row) Idealized arrangements of NPs at 30 and 120 °C that were used to model UV–vis with a brief description of plasmonic coupling within that arrangement. S-S and T-T refer to side-to-side and tip-to-tip coupling between NRs, respectively. More details on the UV–vis modeling are given in Figure 4 and Supplementary Note 2. (4th row) λ_max_ values represented on a common wavelength axis to depict the relations of λ_max_ and Δλ_max_ on thermal switching.

To investigate the temperature-dependent optical properties of the reversibly reconfigurable systems, we performed far-field UV-–vis spectroscopy analysis of NS, NR-L, NR-NS-L1 and NR-NS-L2 films at 30 and 120 °C. Spectra and λ_max_ values are shown in Figure 3 in the top and bottom rows, respectively. The NS system exhibited a broad plasmonic peak with λ_max_ values of ~540 and 520 nm at 30 and 120 °C. The observed Δλ_max_ (20 nm blue shift) can be attributed to the increase in the nearest neighbor distance between NPs from ~4.4 to 6.8 nm, which weakens the coupling between NSs (Figure 1f). It is worth noting that the intrinsic weakness of NS plasmonic properties translates to wide and relatively weak absorption bands, limiting the Δλ_max_ [23] and highlighting the need to prepare reconfigurable systems made of anisotropic NPs with stronger plasmonic properties.

Detailed analysis of UV–vis spectra of NR-based systems is more demanding than that of NSs. One reason is complex couplings in NRs assemblies observed for transverse and longitudinal plasmonic bands; for example, close side-to-side (S-S) or tip-to-tip (T-T) arrangement of NRs will cause a blue or red shift of the signal, respectively [24]. The second reason is the possible variation of structural parameters of the assemblies over bulk scale, e.g., at domain boundaries [23].

For the above-given reasons, finite-difference time-domain (FDTD) modeling was performed to explain the experimental data obtained for four types of arrangements of NR-based systems. Details on the symmetry and periodicities of these arrangements can be found in Supplementary Note 2.

**Figure 4:**
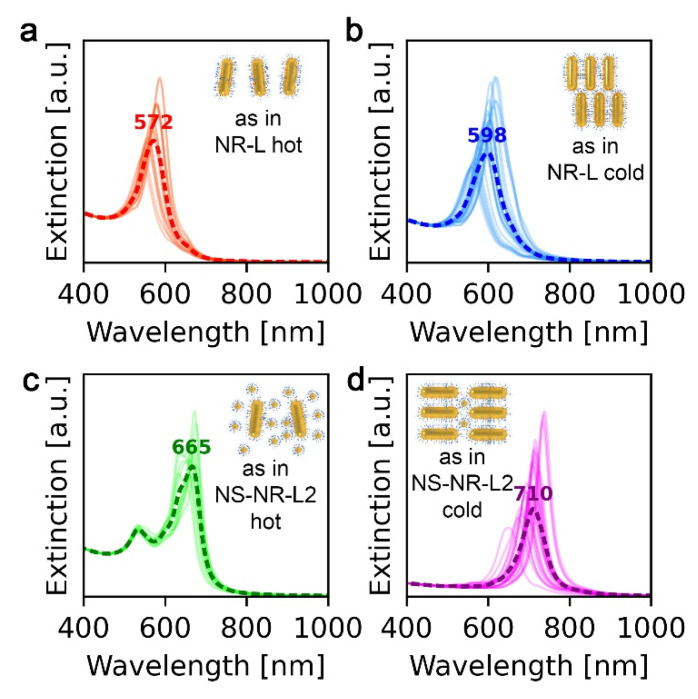
Results of FDTD simulations. (**a**) NRs arranged in a single layer, loosely packed. This simulation corresponds to UV–vis spectra of NR-L and NR-NS-L1 materials acquired at 120 °C (shown in Figure 3). (**b**) Multiple layers of NRs with the noncollinear arrangement of NRs in neighboring layers. This simulation corresponds to UV–vis spectra of the NR-L system acquired at 30 °C and partially corresponds to UV–vis spectra of the NR-NS-L1 system acquired at 30 °C. (**c**) NRs with above 10 nm side-to-side distance and abundant NSs, this simulation corresponds to UV–vis spectra of the NR-NS-L2 system acquired at 120 °C. (**d**) Multiple layers of NRs with the colinear arrangement of NRs in consecutive layers. NSs are present in between NRs. This simulation corresponds to UV–vis spectra of the NR-NS-L2 system at 30 °C.

UV–vis spectra of the NR-L system at 30 and 120 °C revealed extinction peaks at 584 and 564 nm, respectively (Figure 3). To explain the λ_max_ value at low temperature, we hypothesized that the domains of unidirectionally aligned layers of NRs allow for strong S-S coupling, as well as moderate T-T coupling. The latter is moderate, as NRs in consecutive layers are not collinear, as shown in Figure 4b [25]. At 120 °C, nonstacked, loosely formed layers of NRs are present (Figure 2b and Figure 4a), which limits the possibility of T-T coupling, resulting in the blue shift of the plasmonic band. All in all, although the NR-L material exhibits stronger plasmonic properties compared to NSs, the thermal λ_max_ shift is of a comparable magnitude.

Notably, for NR-NS-L1, doubling of the Δλ_max_ was observed in comparison to NR-L. At 120 °C the λ_max_ of NR-NS-L1 (~560 nm) is similar to that of NR-L (~564 nm), indicating the analogous arrangement of NRs in both samples. We can thus conclude that spherical nanocrystals, in the 5:1 ratio, have a limited effect on the plasmonic properties of the assemblies at elevated temperatures. Conversely, the plasmonic band of NR-NS-L1 at 30 °C is centered at ~600 nm, red shifted in comparison to the NR-L system (when no spherical NPs are present). This difference suggests an increase of T-T coupling between the NRs due to the presence of NSs. However, to fully explain the role of NSs, we will focus on the sample with higher content of NSs (NR-NS-L2), making it easier to unveil the NS/NR interactions.

At 30 °C, the NR-NS-L2 exhibits λ_max_ at ~710 nm, red shifted in comparison to NR-NS-L1, suggesting a stronger influence of T-T coupling on the λ_max_. As mentioned earlier, TEM analysis of the NR-NS-L2 sample allowed us to note that the presence of NSs supports the collinear arrangement of NRs (Figure 2d). We can thus conclude that collinearly arranged NRs exhibit stronger T-T coupling. Simulation of the optical response of collinearly arranged NRs confirmed this view (Figure 4d). We can use this knowledge to explain the difference between λ_max_ for NR-NS-L1 and NR-NS-L2—the lower amount of NSs in NR-NS-L1 causes only partial colinear arrangement of NRs, limiting the effect of T-T coupling.

Heating the NR-NS-L2 to 120 °C results in a blue shift of the signal which suggests that T-T coupling strength is weakening. However, in comparison to NR-NS-L1, the λ_max_ is redshifted (~650 vs. ~550 nm). We assume that after melting NSs intercalate in between NRs (Figure 2e). The higher content of NSs, the longer distances between rods and the lower S-S coupling, explaining the difference between NR-NS-L1 and NR-NS-L2. FDTD simulations of this arrangement were in agreement with the observed optical properties of the NR-NS-L2 sample at 120 °C (Figure 4c).

## 4. Conclusions

In summary, the work presented here reveals a facile and robust strategy for fabricating periodic binary films of Au nanorods/nanospheres exhibiting a reversibly reconfigurable structure and controllable plasmonic properties. In this method, the dynamic tunability of NP arrangement is achieved by the melting/freezing of NPs and unbound fractions of LC ligands, enabling reversible transformation between ordered and disordered structures. Combined SAXRD, TEM and FTDT modeling revealed the tendency of unary NR and binary NR-NS assemblies to adopt a 2D layered structure and indicated the vital role of NSs in controlling the plasmonic properties of the binary system by regulating NR coupling. In particular, the evidence suggests that increasing NS content forced NRs to adopt a collinear arrangement and thus increase the T-T coupling of NRs at room temperature while minimizing NR-NR coupling at high temperatures. The latter suggests that NSs exhibit a solvent-like behavior. The work presented here reveals the way to combine thermoresponsiveness of LC-like ligands and plasmonic coupling of binary assemblies of plasmonic NPs, providing a useful methodology for achieving switchable nanomaterials with complex plasmonic properties. Moreover, the presented ability to produce ordered active binary NP assemblies can be a starting point for further research, expanded to include different NP shapes or to encompass long-range anisotropic NP orientation, leading to polarization-dependent optical interactions.

## Data Availability

The data presented in this study are available in article and Supplementary Material.

## Supplementary Materials

The following are available online at https://www.mdpi.com/article/10.3390/nano11092296/s1, Supplementary Note 1: XRD analysis of NS and NR systems, Supplementary Note 2: Plasmon coupling in the obtained assemblies, Figure S1: Organic synthesis, Figure S2: Additional XRD data, Figure S3: Additional TEM images of NS assemblies, Figure S4: NR distance analysis, Figure S5: Additional TEM images of NR-based binary assemblies, Figure S6: TEM images showing different NR-NS arrangements.
