# Supplementary material for: Active Plasmonics with Responsive, Binary Assemblies of Gold Nanorods and Nanospheres
Source: Nanomaterials (Basel). 2021 Sep 3;11(9):2296. doi: 10.3390/nano11092296 (PMC8465109; doi:10.3390/nano11092296)
Supplement: Supplementary file 1 [file nanomaterials-11-02296-s001.zip › nanomaterials-1322590-supplementary.pdf]

nanomaterials

Supplementary Materials

Active Plasmonics with Responsive, Binary Assemblies of Gold Nanorods and Nanospheres

Piotr Szustakiewicz, Natalia Kowalska, Maciej Bagiński and Wiktor Lewandowski *

Faculty of Chemistry, University of Warsaw, 1 Pasteura st., 02-093 Warsaw, Poland; p.szustakiewicz@chem.uw.edu.pl (P.S.), n.kolsut@student.uw.edu.pl (N.K.), mbaginski@chem.uw.edu.pl (M.B.)

* Correspondence: wlewandowski@chem.uw.edu.pl

Supplementary note 1: XRD analysis of NSs and NRs systems.

The system made of spherical gold nanoparticles covered with a monolayer of dodecanethiol and liquid crystal-like (LC-like) ligand presented here behaves similarly to other systems of spherical nanoparticles covered with a mixed monolayer of ligands described in our previous research. [1,2] At low temperature, due to interactions between aromatic cores of the L ligands, the organic coating of NPs adopts an oval shape (due to bundling of LC-like ligands). Thus, NPs adopt a body-centred tetragonal (bct) arrangement. After increasing the temperature above ~90 °C, the aromatic interactions are not strong enough to maintain the anisotropic shape of NPs organic coating, which results in an overall spherical shape of the nanoparticle, leading to an isotropic arrangement.

In the case of the NRs system, the ratio of LC-ligands to metal is much lower than in the case of NSs, due to the larger size of NRs in comparison to NSs. Thus, ligand bundling is not playing a role in defining the arrangement of the NRs, thus NRs form a close-packed structure. This structure is stable within the tested thermal range, as evidenced by the temperature evolution of the small-angle x-ray diffraction (SAXRD) diffractograms (**Figure 1g** in the main text).

Supplementary note 2: plasmon coupling in the obtained assemblies

To explain the peak positions of the NR-based assemblies described in this work, Finite Domain Time Difference modelling was performed using Lumerical software. Due to some degree of variation and/or uncertainty in structural parameters of the assemblies (e.g. different NR side-to-side distances, as seen in Figure S4) we decided to perform a series of simulations, varying selected parameters of each structure. SAXRD and TEM data were used to determine the mean structural parameters of the assemblies. For all NRs-based structures, at low temperatures, the tip-to-tip distance between NRs along long NR axis as well as the side-to-side distance was 3 ±2 nm. The number of NRs was varied between 3 and 7 within a single layer. A total of 2 to 5 layers was simulated. To reproduce high-temperature cases, single layer assemblies were simulated. In the case of NR-L, the side-to-side distance was 5 ± 2 nm. In the case of NR-NS-L2, the side-to-side distance was 10 nm. In the latter, we also included from 10 to 35 randomly placed spherical NPs, with the minimal distance between two NPs of at least 2 nm.

To simplify the explanation of the plasmonic peak positions, the *strongest/medium/weakest* quantification was introduced in Figure 3 in the main text to describe the differences in coupling strength in different assemblies. Side-to-side (S-S) and tip-to-tip (T-T) coupling regimes were treated separately.

The side-to-side designation relies solely on the side-to-side distance. Namely, for all assemblies of NRs at low temperature, this distance is the lowest. Thus, the side-to-side coupling is marked as the *strongest*. The term *medium* side-to-side coupling was assigned for NR-L and NR-NS-L1 at high temperatures, as the side-to-side distance is greater than

at low temperatures. The *weakest* side-to-side coupling was assigned to NR-NS-L2 at high temperatures, since of NSs in this system further increases inter-NRs distance.

In the case of the tip-to-tip coupling, the term *strongest* is used when the NRs are collinearly arranged in consecutive layers, the term *medium* when NRs in consecutive layers are not collinearly arranged, while the term *weakest* was used when NRs do not form a layered structure (a single layer of NRs was simulated).

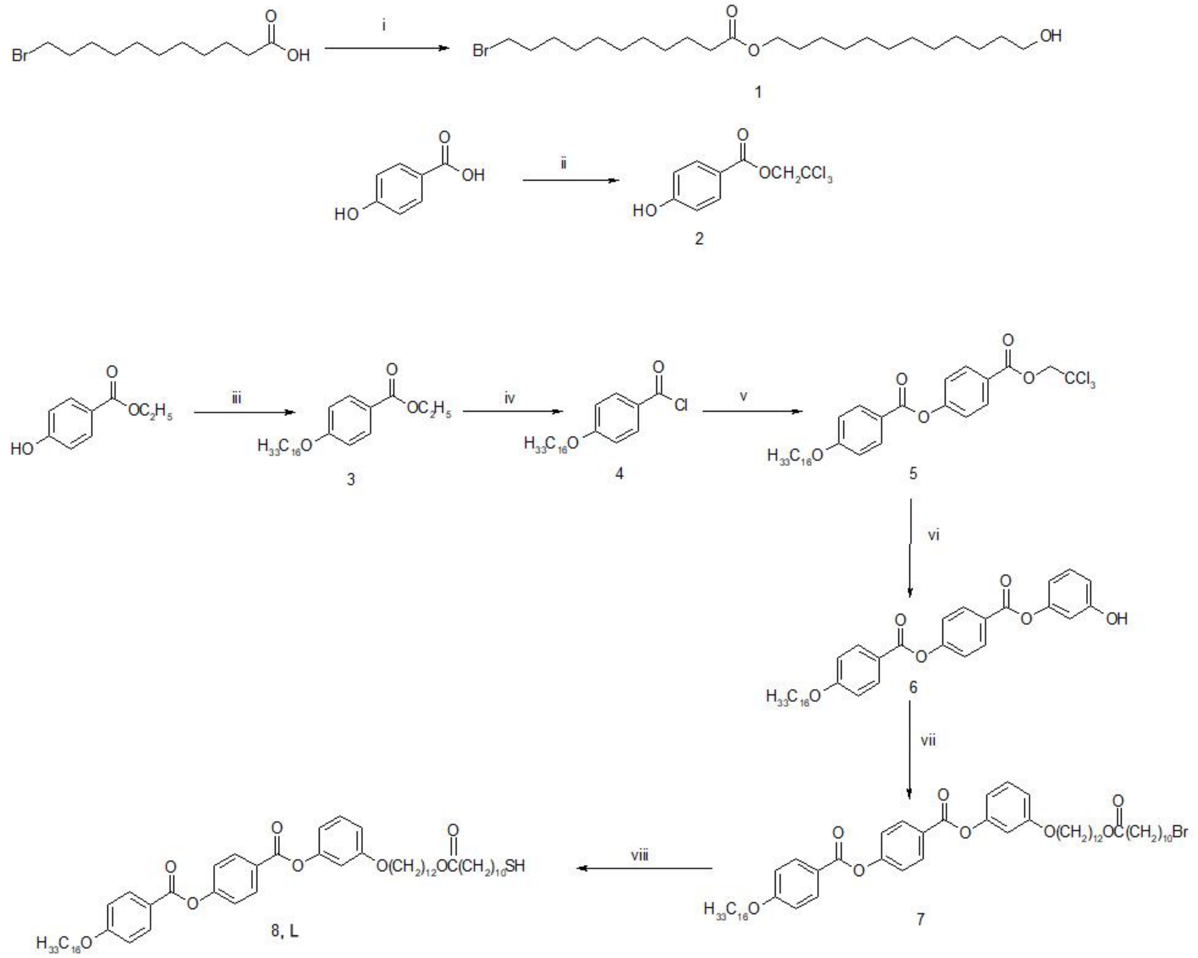

Figure S1. Synthetic route for the preparation of the liquid crystal-like ligand L. Reagents and conditions: (i) a. oxalyl chloride, DMF (dimethylformamide), DCM (dichloromethane); b. 1,12-dodecanediol, pyridine, DMAP (4-dimethylamino-pyridine), THF (tetrahydrofuran); (ii) 2,2,2-trichloroethanol, $NaHCO_3$; (iii) cetyl alcohol, PPh_3, DIAD (diisopropyl azodicarboxylate), THF, ultrasounds; (iv) a. KOH, ethanol, rfx.; b. oxalyl chloride, toluene, rfx.; (v) compound 2, TEA (Triethylamine), DMAP, THF, rfx.; (vi) a. oxalyl chloride, toluene, rfx.; b. resorcinol, TEA, DMAP, THF; (vii) compound 1, PPh_3, DIAD, THF, ultrasounds; (viii) HMDST (bis(trimethylsilyl)sulfide), TBAF (tetra-n-butylammonium fluoride), THF.

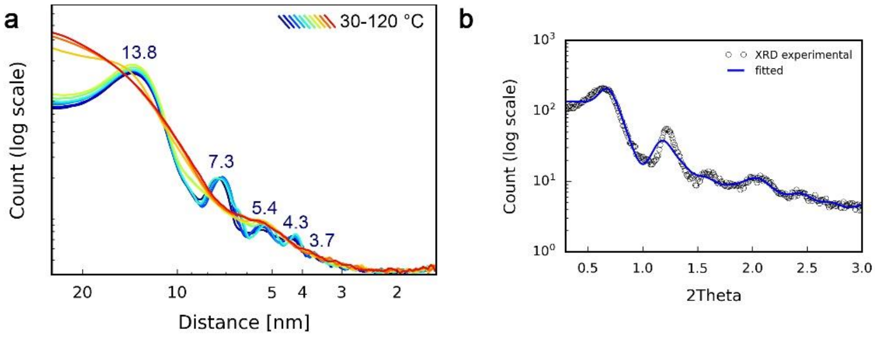

Figure S2. **(a)** Temperature-dependant SAXRD patterns of binary systems comprising NSs, NRs and unbound ligand at NS:NR ratio of 5:1 (NR-NS-L1). (**b**) An overlay of experimental, low-temperature structure diffractogram of NR-NS-L2 (as shown in main text Figure 2f) and fitted hexagonal phase with *a*~14.1 nm (signals: 010 - 13nm, 110 – 7.5 nm, 020 – 6.5nm,

210 – 4.9 nm, 030 – 4.3 nm, 220 – 3.8 nm, 310 – 3.6 nm). We attribute the non-perfect overlay of the fitted signal with the experimental signal at 7.2 nm to NS assemblies (NS layers or phase-separated NSs), since periodicities of ca. 7 nm can be attributed to interparticle distances in NSs assemblies in the isotropic phase (as shown in panel b), suggesting that the presence of NRs in the binary systems and unbound ligands at least partially disrupts the formation of a perfectly ordered body centered tetragonal phase.

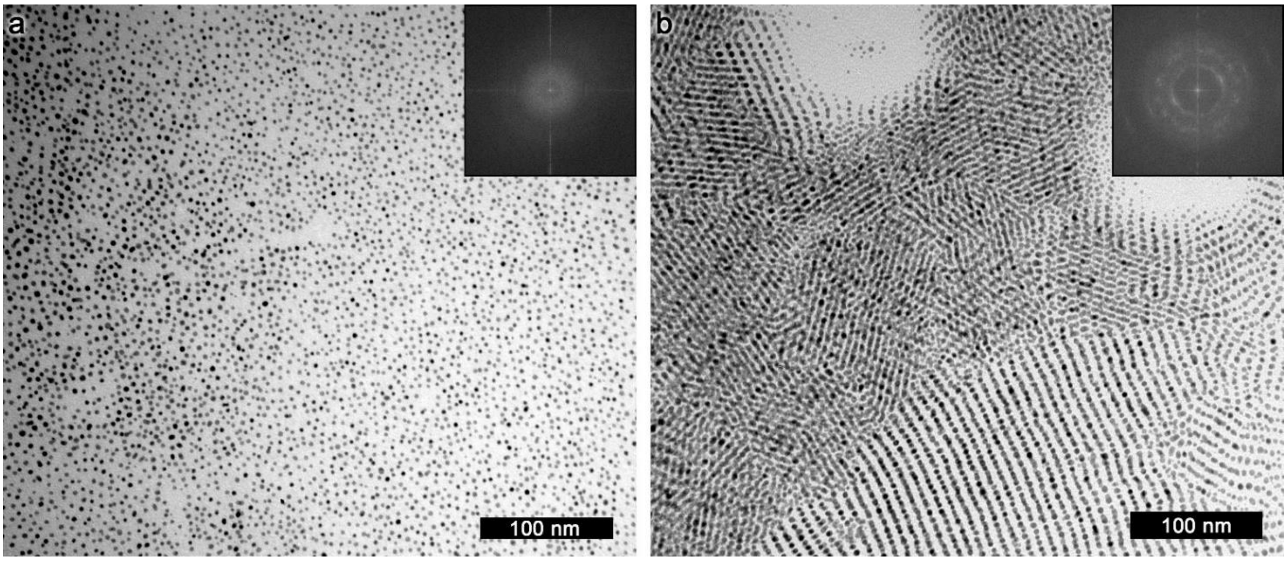

Figure S3. TEM images of Au NSs functionalized with L. (**a**) Sample quenched at 120 °C. (**b**) Sample heated to 120 °C and slowly cooled down to 30 °C . Insets show corresponding fast Fourier transform (FFT) images.

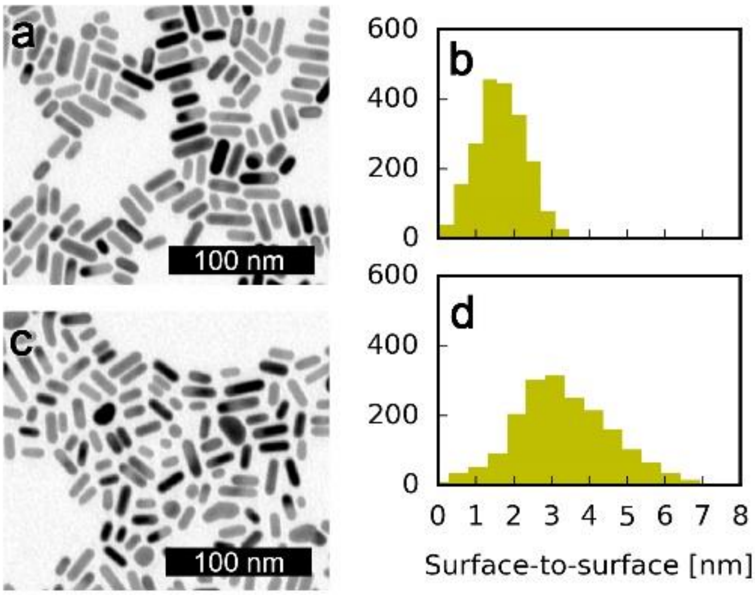

Figure S4. TEM images along with histograms of the surface-to-surface distance between neighbouring NRs. (**a**,**b**) Pure NRs. (**c**,**d**) NR-L sample.

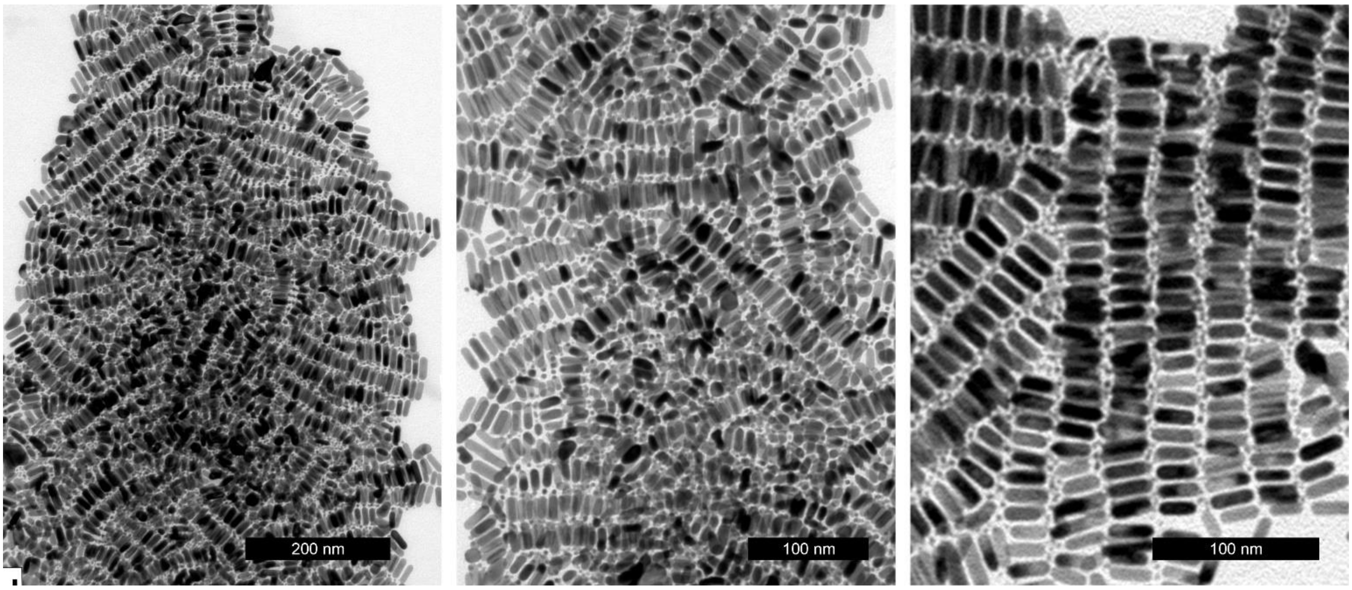

Figure S5. Additional TEM images of NR-NS-L2 sample with different magnifications showing alternating layers of NRs and NSs.

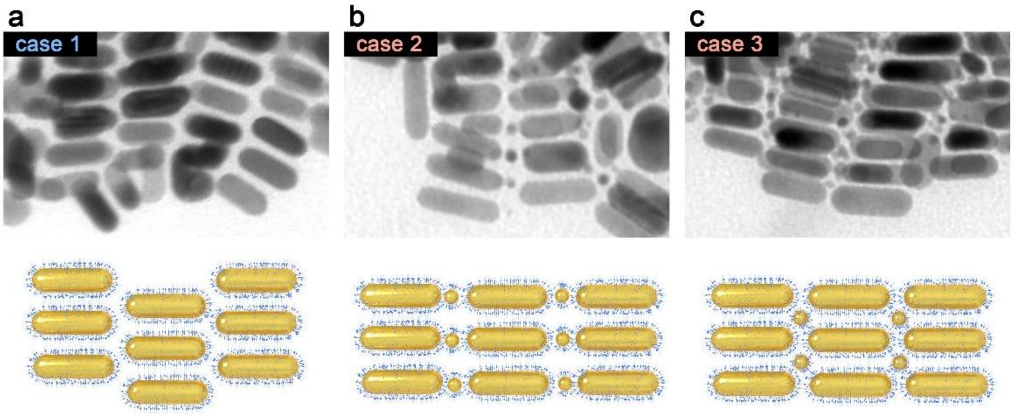

Figure S6. Three distinct cases of AuNR/AuNS arrangement with either non-collinear (**a**) or colinear arrangement of NRs (**b**,**c**). For each of the three cases TEM images (top) and graphical representation (bottom) are shown.

Bibliography:

1. Lewandowski, W.; Fruhnert, M.; Mieczkowski, J.; Rockstuhl, C.; Górecka, E. Dynamically self-assembled silver nanoparticles as a thermally tunable metamaterial. *Nat. Commun.* **2015**, *6*, doi:10.1038/ncomms7590.
2. Lewandowski, W.; Łojewska, T.; Szustakiewicz, P.; Mieczkowski, J.; Pociecha, D. Reversible switching of structural and plasmonic properties of liquid-crystalline gold nanoparticle assemblies. *Nanoscale* **2016**, *8*, 2656–2663, doi:10.1039/c5nr08406g
